# Sverdrup-Henson crater: A candidate location for the first lunar South Pole settlement

**DOI:** 10.1016/j.isci.2023.107853

**Published:** 2023-09-09

**Authors:** Giovanni Leone, Caitlin Ahrens, Jarmo Korteniemi, Daniele Gasparri, Akos Kereszturi, Alexey Martynov, Gene Walter Schmidt, Giuseppe Calabrese, Jari Joutsenvaara

**Affiliations:** 1 Instituto de Investigación en Astronomía y Ciencias Planetarias, Universidad de Atacama, Copiapó 153000, Chile; 2 NASA Goddard Space Flight Centre, Greenbelt, MD, USA; 3 Arctic Planetary Science Institute (APSI), Rovaniemi, Finland; 4 Research Centre for Astronomy and Earth Sciences, Konkoly Thege Miklos Astronomical Institute, Budapest, Hungary; 5 Maana Electric, Dubai, United Arab Emirates; 6 Department of Science, Roma Tre University, Rome, Italy; 7 International Research School of Planetary Sciences (IRSPS), Universitá“D’Annunzio”di Chieti e Pescara, Chieti, Pescara, Italy; 8 Kerttu Saalasti Institute, University of Oulu, Oulu, Finland; 9 Underground Science, Research & Development Centre Callio Lab, Pyhäjärvi, Finland

**Keywords:** Space sciences, Astronomy, Multidisciplinary design optimization

## Abstract

Robotic and manned exploration of the Moon is the next target in Solar System exploration. The availability of *in situ* resources such as water ice, iron oxides, helium-3, and rare earth elements, combined with permanently sunlit areas, provides the opportunity for the first settlement, either human or robotic, on the Moon. We used several selection criteria (abundance of water ice, the slope of terrain, usable energy sources, communications, and base expandability) to identify a suitable area for a future base in the southern polar crater Sverdrup-Henson. Due to the higher abundance of water ice, we found that the Sverdrup-Henson site is better suited to host a base than the nearby craters de Gerlache and Shackleton. The crater floor is partly in permanent shadow and exhibits numerous signatures of water ice. Since water ice is essential for rocket fuel production and human survival, its presence is necessary for a first settlement. Sverdrup-Henson has a flat floor ideal for building and safe traversing, is accessible from the surrounding intercrater plains, and has nearby locations suitable for communications and solar power production. Thus, the Sverdrup-Henson site holds great potential for future missions. We propose further exploration of this area through *in situ* measurements to better constrain available resources.

## Highlights

• Sverdrup crater is a suitable place for a first lunar settlement
• The PSR of Sverdrup contains more water detections than its neighbor craters
• Sverdrup crater is easily accessible and has enough space to expand the settlement

## Graphical abstract

**Figure:**
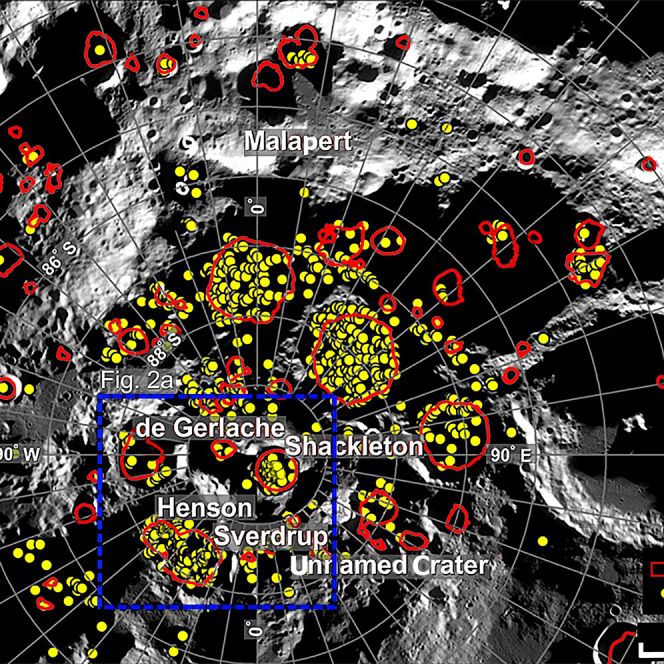

## Introduction

**Figure 1:**
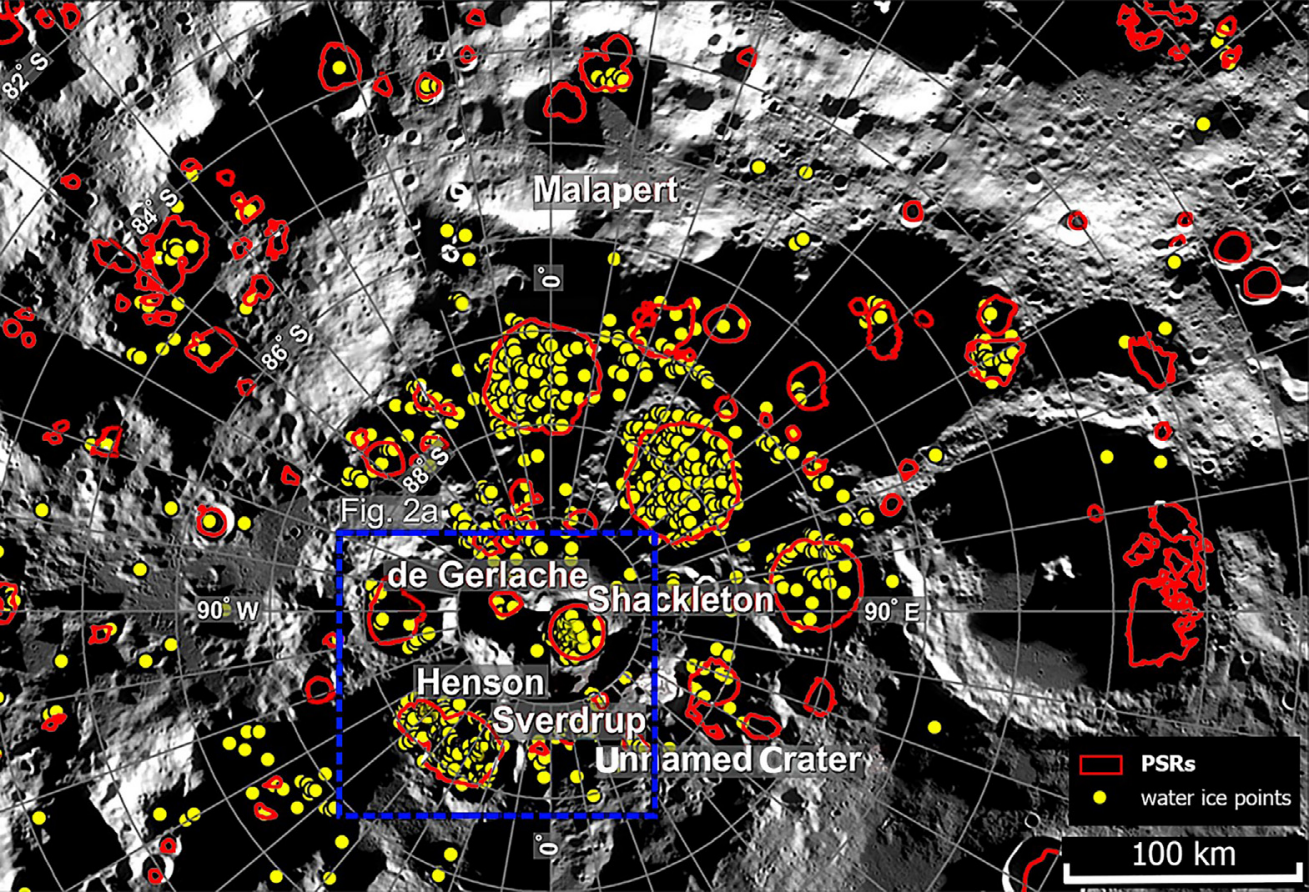
Regional map of the study area (blue dashed line), indicating the craters Sverdrup, Henson, Unnamed Crater, de Gerlache, Shackleton, and Malapert Massif The red lines enclose PSRs.^7^ The yellow dots represent the ice exposure points as constrained by the Chandrayaan-1 M^3^ instrument (Li et al.^8^). Basemap: LRO LROC-WAC Global Mosaic at 100 m/pixel.

The lunar South Pole (Figure 1) is currently considered one of the most intriguing study regions for a first lunar base.^1^^,^^2^^,^^3^ A lunar base would be a significant step for both long-term robotic and human missions, as well as a possible future launching pad to Mars.^4^^,^^5^^,^^6^

The primary criteria to take into consideration for the selection of an exploration and settlement can be summarized as follows.^9^^,^^10^

1. The availability of water ice per km^2^ of floor area and mineral resources, at least 0.2 water detections per km^2^, would make the site convenient. Water is an essential resource for the extraction of hydrogen and oxygen for rocket propellant^11^ and other uses (i.e., drinking water, irrigation of hydroponic cultivation, etc.) in human-related activities (i.e., Lewis^12^^,^^13^; Li et al.^14^). Carbon dioxide (CO_2_) molecule is also a valuable resource for C and O and a proponent of chemical fuels.^15^ CO_2_ can be found as a frozen volatile at the lunar South Pole as cold traps.^16^ Oxygen can also be extracted from iron oxides through a process of thermal reduction at high temperature.^17^^,^^18^ Frozen volatile investigations are also the main objectives with the future Artemis III mission^19^^,^^20^ and other lunar-specific missions, such as Lunar Trailblazer^21^ and Volatiles Investigating Polar Exploration Rover (VIPER) (Colaprete et al.^22^; Stopar and Shearer^23^).
2. The slope of the terrain. Although the ideal base site would have a flat surface, even moderate slope angles may still be considered reasonably safe, depending on the task.^10^ A slope of 7° would allow spacecraft landing, while mobile surface operations (i.e., astronaut-only extra-vehicular activities [EVAs]) are rather safe on up to 15° slopes.^9^^,^^10^ The Apollo 15 crew reported an excellent performance of the lunar rover vehicle (LRV) on slopes of 12°.^24^ Another study considered suitable-sites-only locations with less than 5° of slope in at least a landing ellipse of 1 km of diameter, presence of permanently shadowed regions (PSRs), and the peak of light (POL).^25^ However, this study does not consider Sverdrup among the regions of interest, although it meets the mentioned slope and solar illumination criteria, while our study does.
3. Usable energy source. Presence of sufficient sunlight (>50% of the lunar day) for power generation by solar panels.^26^
4. Communications link. (Semi-)continuous visibility of Earth is essential for the continuity of communication, remote control of robotic operations, and crew safety and morale. Such a direct line-of-sight connection would be the most robust scenario. Although various options may be considered, such as peak-to-peak antenna relay chains, or a system of lunar polar relay satellites, we focus on the primary mechanism of direct connection from Earth.
5. Designated area for base operations and expansion. The base site should have enough area for regular ground operations, say 81 km^2^, which includes part of the PSR, and the potential for future expansion.
6. Scientific interest. The duration of the stays and the processing operations of the lunar materials to develop the settlement into a future base will make it possible to acquire samples from the highlands to understand the formation and evolution of the primary crust of the Moon, similarly as done by the Chang’e 5 mission in the maria of Oceanus Procellarum (Li et al.^27^).

The lunar South Pole has already been studied as a possible strategic area, particularly around Malapert Massif (aka Mount Malapert, Malapert Mountain, or Malapert Mons).^1^^,^^28^ This is an interesting site, despite it is characterized by 6°–15° mean slopes considered on Lunar Orbiter Laser Altimeter (LOLA) spatial resolution (about 16 m/pixel),^29^ and it is suitable for solar energy production being exposed to sunlight for 93% of the lunar year.^1^ However, Malapert Massif misses the important criteria such as nearby *in situ* resources and expansion possibilities (see sections Methods for the assessment of landing site suitability criteria and Results for further details). Shackleton crater has also been considered a suitable place for a settlement due to its favorable illumination conditions.^30^ Previous works elaborated on the possible ways to exploit solar energy but mostly focused on technical issues related to the application of electric microgrids^31^ or analysis of illumination conditions for the placement of reflectors aimed at providing continuous solar power at Shackleton crater.^32^ These studies did not have a detailed analysis of slope and *in situ* resource utilization (ISRU). Another study analyzed slope, energy, and ISRU but has not mentioned Sverdrup-Henson.^25^ Our study goes beyond this because it also identifies a suitable site for the first settlement on the Moon.

**Figure 2:**
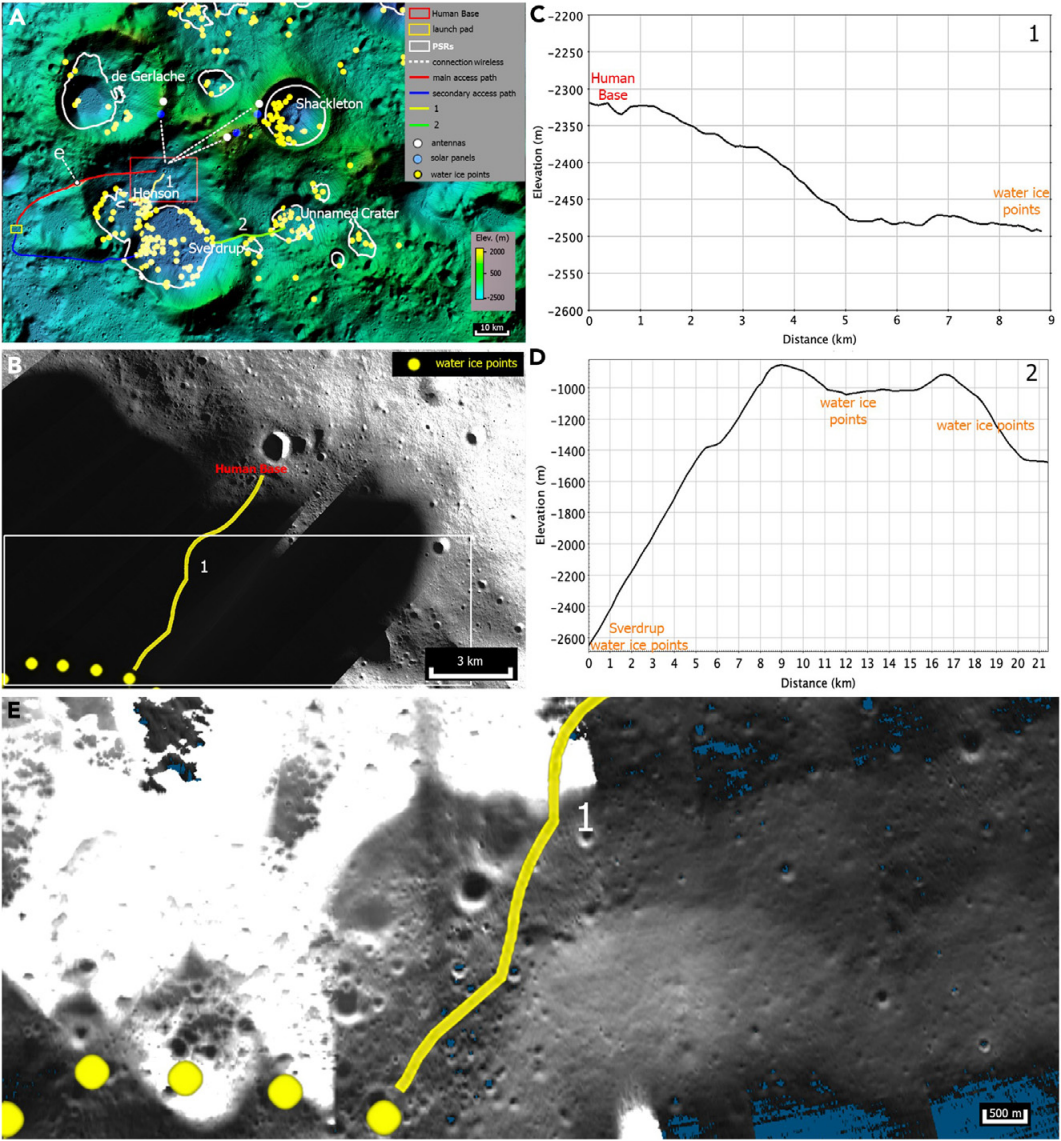
Water resources and detailed topography of Sverdrup crater (A) Detailed color Hillshade LOLA DEM South Pole map (over the LRO LROC-WAC Global Mosaic at 100 m/pixel) of the location of the proposed human (or robotic) base/settlement (red square), the white dashed lines indicate the lines of sight and wireless connection from the solar panels and antennas located on the surrounding hills/crater rims to the base, the white contour lines indicate the PSRs. (B) Blowup of the base area with LRO LROC-NAC mosaic at 13.75 m/pixel, the yellow line indicates the shortest path to the PSRs where the ice exposure points (yellow dots) are located. (C) topographic profile along the yellow path shown in panel B. (D) topographic profile along the green path between Sverdrup and the Unnamed Crater shown in panel A. (E) Additional blow-up of the white rectangle shown in panel B, LROC NAC Buffered PSR Mosaic (v2) South. Resolution: 9.86 m/pixel.

Based on these criteria, we identified a study area centered on the crater Sverdrup (88.5°S, 152.0°W) and an older, partly underlying crater named Henson (88.5°S, 129.6°W)^33^ (Figure 1). To set up a permanent human base, we propose a place located within the Sverdrup-Henson crater (red square in Figure 2A). Both crater floors exhibit PSRs with maximum temperatures of 110 K,^34^ which is important for direct access to water from the base. The illuminated terrain visible in Figure 2B is all available to deploy the base, and we added the shortest path (yellow line) toward the PSRs, which is further indicated by a topographic profile in Figure 2C. PSRs are never directly illuminated by sunlight and act as cold traps for ice and other volatiles (Figures 2A and 2B).^35^^,^^36^ Other PSRs around the South Pole are located within impact craters with estimated ages of over 3 Ga.^37^ The rim between Henson and de Gerlache has illuminated areas for most of the lunar year.^38^^,^^39^ Throughout the study, we compare the Sverdrup-Henson site with the nearby craters, Shackleton (89.4°S, 129.4°E) and de Gerlache (88.5°S, 87.1°W), as well as the previously proposed lunar base site, Malapert Massif (85.8°S, 3.8°E).^1^^,^^28^ We selected these particular craters for this study because they are the closest to the South Pole of the Moon, which is important for the illumination conditions and proximity to PSRs.

### Other suitable places for a lunar base settlement

The possible presence of water ice at both lunar poles within the PSRs was first detected by the Clementine and Lunar Prospector missions.^40^ However, a few studies have been made regarding the amount or feasibility of water ice extraction on the Moon.^41^ All estimates are based on the spectral signatures of the orbiting instruments except for the crater Cabeus where Lunar Crater Observation and Sensing Satellite (LCROSS) impacted, thus providing a first *in situ* sampling of a PSR.^42^ The PSRs at the lunar South Pole are estimated to have an abundance of ∼2.9 × 10^12^ kg of water ice,^43^ suggesting a sufficient volume to support the daily activities of an initial future human base. Estimates of the water need for an astronaut vary from 1 or 2 L^44^ to 3.5 L^45^ per day.^46^

Based on the Lunar Reconnaissance Orbiter (LRO) Lyman Alpha Mapping Project (LAMP) data,^47^ the PSRs around the lunar South Pole were estimated to contain 0.1–10 wt. % of water ice. The amount depends on the extent of the specific PSR and the assumed type of exposed surface material. Similarly, the LCROSS data indicated that the floor deposits within the near-polar PSR crater Cabeus contain 4–8 wt. % of water ice^48^ with a mean concentration of 5.6 ± 2.9% by mass.^49^ Later, the SOFIA (Stratospheric Observatory for Infrared Astronomy) telescope identified water molecules (stored in intergranular spaces) and hydroxyl ions also on a sunlit lunar surface, namely on the floor of the crater Clavius (N-S extent 54.82–62.43°S). However, they were found to contain a much lower amount of water, less than 0.04 wt. % (100–400 μg/g).^50^

Another fundamental factor is the presence of available mineral resources for construction and technological equipment, i.e., ISRU. These include iron and titanium oxides^51^ and rare earth elements.^52^^,^^53^ These materials are the most abundant in the Oceanus Procellarum KREEP Terrane (PKT), in particular the eastern part of the Em4 (mare basalt) geological unit near the Mairan domes, recently sampled by the Chang’e 5 mission.^54^^,^^55^ This area is also important for the rare earth elements, up to 4.6 wt. % yttrium and up to 0.25 wt. % neodymium, as well as our study area in the South Pole region. However, there is not yet evidence anywhere on the Moon of concentrations that could potentially be classified as ore.^53^

The aim of this work is to evaluate and compare potential base sites and find an ideal location for a first exploration mission for a future lunar base in which a safe landing area, solar illumination, and access to permanently shadowed areas are present all together. This will further constrain the value of each crater within the lunar South Pole and further our appreciation of the region in general. In this way, a basic framework for future missions is provided.

### Methods for the assessment of landing site suitability criteria

#### Datasets implementation

**Figure 3:**
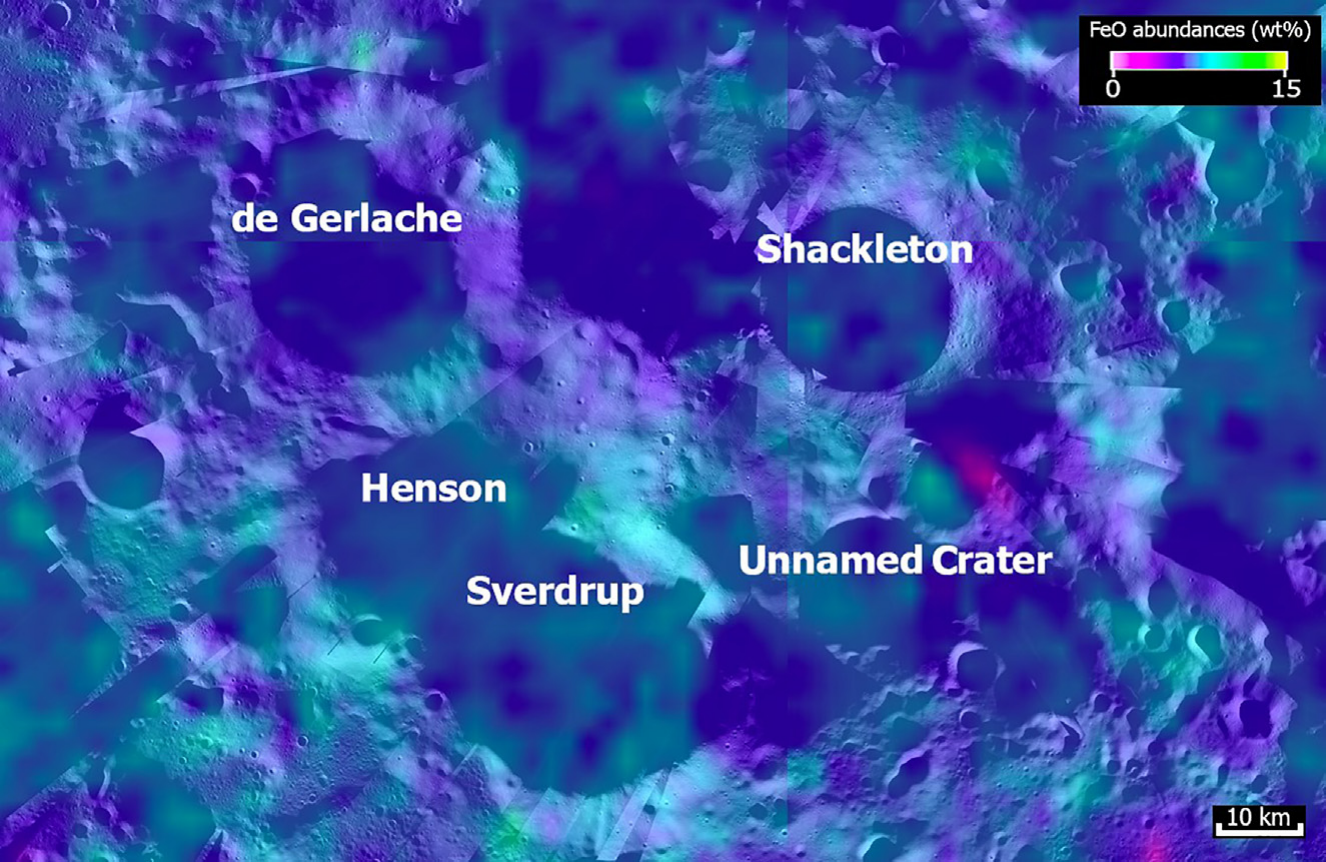
Map of iron oxide abundance Regional map of the polar iron oxide (FeO) abundance as derived by the interpolation between reflectance data at 1064 nm from the Lunar Orbiter Laser Altimeter (LOLA) and the 955.5/752.8 nm reflectance ratio from the Kaguya Spectral Profiler (SP) (from Lemelin et al., 2017). Basemap: LRO LROC-WAC Global Mosaic at 100 m/pixel.
e. The colorized LOLA slope map at 16 m/pixel resolution,^63^ as shown in Figure 4. We also used the slopes from the Kaguya-LOLA combined digital terrain model (SLDEM2015)^70^ to determine the small-scale details on slopes.

Building on the landing site characterization criteria introduced in the previous section,^9^^,^^10^ we conducted a preliminary survey using the datasets also available on the online tool NASA Moon Trek,^56^ Java Mission-planning and Analysis for Remote Sensing (JMARS),^57^ and other publicly available data sources like the Permanently Shadowed Regions Atlas (PSRA).^58^ We imported the datasets listed below and analyzed them using ArcGIS. Measurements of areas, distances, elevations, and slopes (results for which are shown in sections Craters in the area of study and site location and Slope), as well as analysis of accessible sites of maximum illumination and Earth visibility (sections Illumination conditions and Communications with Earth), were made using the Lunar Reconnaissance Orbiter Camera (LROC) Quickmap online tool.^59^ Ready-to-use map of M^3^ water detections is taken from the MoonTrek website.^56^ Datasets used in this study are listed in the following.

a. The LROC Wide Angle Camera (WAC) Mosaic at 100 m/pixel^60^ and the Narrow Angle Camera (NAC) at 0.5–2.0 m/pixel.^61^
b. The shapefile layer of the LOLA for the PSRs,^7^ as shown in Figures 1 and 2.
c. The shapefile layer of the spectroscopic detections of water ice (280 m/pixel), as constrained by the Chandrayaan-1’s Moon Mineralogy Mapper (M^3^) (Li et al.^8^), is also downloadable from institutional sites such as the Planetary Data System (PDS)^62^ as well as the LOLA,^29^^,^^63^ the Diviner Lunar Radiometer Experiment,^64^ and the Lyman Alpha Mapping Project (LAMP)^49^^,^^65^ instruments (Figures 1 and 2). In the specific case of the M^3^ water detections, each dot of the water detections represents an M^3^ pixel, roughly a ∼280 m × 280 m area of the ground, which represents a separate detection from the other points (Li et al.^8^).
d. The map of polar iron oxide (FeO) abundance at 100 m/pixel (Figure 3), as derived by the interpolation between reflectance data at 1,064 nm from the LOLA and the 955.5/752.8 nm reflectance ratio from the Kaguya Spectral Profiler (SP),^66^^,^^67^^,^^68^^,^^69^ as shown in Figure 3.

**Figure 4:**
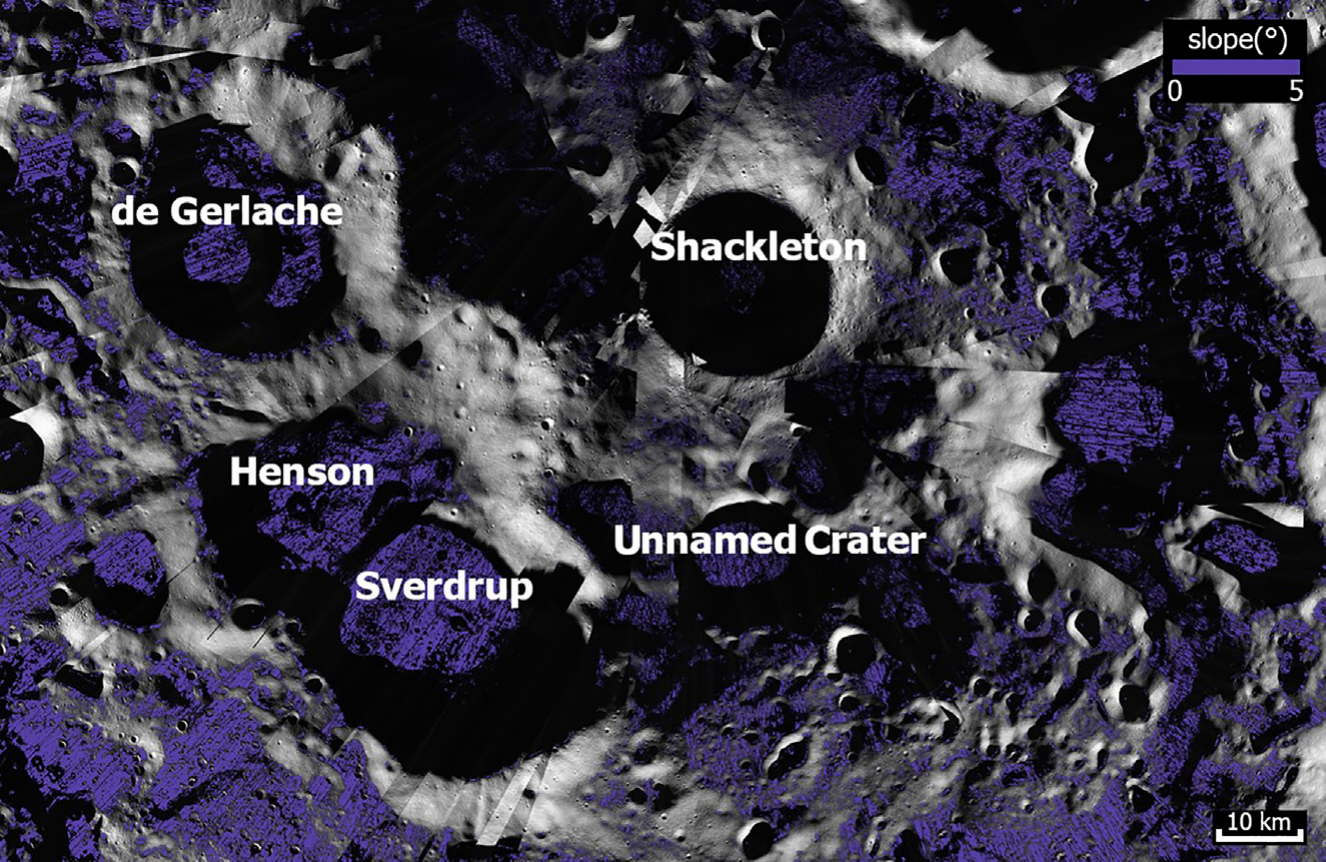
Map of regional slope LOLA slope colorized map. Basemap: LRO LROC-WAC Global Mosaic at 100 m/pixel.
f. Kaguya data combined with LOLA data also gave important indications about the illumination conditions in the South Pole Aitken (SPA) basin^7^^,^^38^^,^^71^ and the illumination map of the Moon’s South Pole at 100 m/pixel (Figure 5).

**Figure 5:**
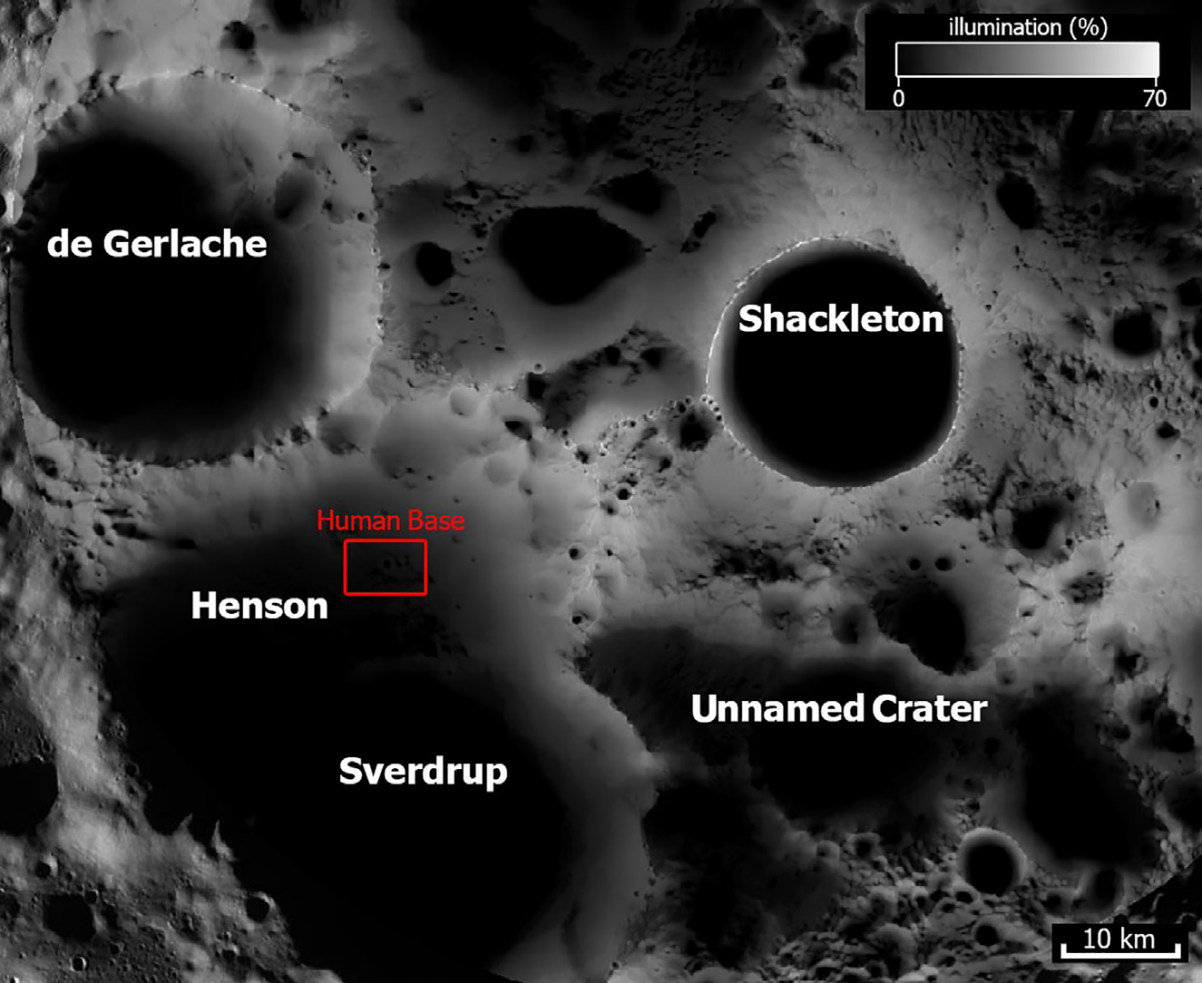
Map of illumination with human base NAC Percentage-Based South Pole Illumination Map (100 m/pixel).

#### Selection criteria

##### Presence of water ice

We studied how the compared sites were proximal to PSRs (see Figure 1) and to the presence of spectroscopic detections of water ice (Figures 1 and 2). A previous study, analyzing three spectral bands at 1.3, 1.5, and 2 μm, respectively,^8^ of Chandrayaan-1 M^3^ infrared spectra, detected the possible presence of water ice exposed on the surface of the lunar south polar region. Another study identified 169 PSRs distributed on 8 craters (Shoemaker, Faustini, Cabeus, Malapert, Nobile, Sverdrup, Wiechert J, and Haworth plus three unnamed craters) located both on the near side (98 PSRs) and on the far side (71 PSRs) of the Moon within 6° of latitude from the South Pole with a total of 820 points of water ice detections.^68^ In the results section (Water ice), we found 230 water ice detections within only 5 craters (Shackleton, de Gerlache, Henson, Sverdrup, and Unnamed Crater), all concentrated in the South Pole area. We counted the number of reported detection points within all the various craters of the lunar South Pole (Figure 2). Based on the number of ice exposure points, we categorized the total occurrence (regardless of the total crater area) into scarce (less than 10 points), sufficient (between 10 and 50 points), good (between 50 and 100 points), and excellent (more than 100 points). However, we will show in the results section (Water ice) how this aspect will not significantly change in terms of the total amount of water ice detections.

##### Mineral resources

The presence of variable resources within the regolith (i.e., iron, nickel, plagioclase, pyroxene, olivine, ilmenite, rare earth elements, and helium-3)^72^ may be used for ISRU activities. The lunar South Pole has a prevailing anorthositic composition, but the presence of FeO means that there are slightly more mafic intrusions other than anorthosite at the regional scale (Figure 3). This is an important criterion for the selection. Among potentially usable minerals, the most abundant in the lunar regolith are plagioclase, pyroxene, olivine, ilmenite, and spinel, followed by native metals like Fe and Ni,^17^ especially for the engineering of using such materials for the construction of a potential human base.^73^ In this study, we based our selection on the available map of the FeO oxides (Figure 3), whose abundance is essential for oxygen extraction. However, the Metalysis-FFC electro-deoxidation process is able to extract oxygen from the lunar regolith and creates some useful metal alloys as a by-product.^74^ Also, extraction of Al from regolith is useful as this metal is easier to work and to lift. It is possible to obtain Al, Si, and Al_5_FeSi by aluminothermic reduction at 980°C for 4 h with chemical components of 79.71 mass% aluminum, 12.03 mass% silicon, 5.91 mass% iron, and 2.35 mass% titanium.^75^

##### Illumination conditions

Ideally, the base site would receive permanent sunlight, but no such areas are on the Moon.^7^ However, there are some places where the illumination percentage is above 80% of the lunar day.^7^ The percentage should be >50% to avoid using nuclear power.^76^ Such an amount of solar radiation would allow both adequate availability of solar energy and favorable thermal conditions for surface operations.^76^

##### Communicating with Earth

The visibility of Earth is important for a constant communications link between the base and the home planet, although more important is the direct line of sight to the network of communication such as the Deep Space Network. At a minimum, at least a fraction of Earth’s disk should be visible from the given location on the Moon.^7^ It was estimated that the Earth disk would be ∼50% visible from the rims of Shackleton and de Gerlache craters;^7^ this was, however, deemed a low percentage of time for adequate communications with the lunar base, and the Malapert Massif (with 93% Earth visibility) was proposed as a site for relay antennas.^77^ During future Artemis activities, technology demonstrations and scientific exploration will be conducted over longer periods (months or years) and will need the Lunar Gateway’s orbit around the Moon via a nearrRectilinear halo orbit (NRHO),^78^ which would be a huge proponent of communication issues for the exploration at the lunar poles.

## Results

### Craters in the area of study and site location

Five craters within the lunar South Pole, Henson, de Gerlache, Sverdrup, Shackleton, and an unnamed crater form the basis of our measurements and comprise the study area. The ∼45 km diameter Henson is the oldest among the studied in this work because the other nearby craters superpose it; its rim is broken by de Gerlache, Sverdrup, and numerous smaller craters. In the remaining segments, Henson’s rim-to-floor height is ∼1.0–3.5 km. Sverdrup is ∼33 km in diameter and has a slightly higher ∼2–4 km rim-to-floor elevation difference, with the highest peaks on the side opposite of Henson. However, the ∼18 km stretch of Sverdrup’s rim between the two craters is anomalous. There, the rim rises only 0.2–0.7 km above Sverdrup’s flat floor (which has an average elevation of −2,880 m and an area of ∼257 km^2^) and stays level with or is at most 0.3 km higher than Henson’s slightly more rugged floor (area ∼222 km^2^, average elevation −2,250 m). The site of interest for the eventual lunar base is a square ∼4.5 × 4.5 km located on the north-eastern side of the Sverdrup-Henson crater at the border between the PSR and the partially illuminated zone (Figures 2A and 2B). We used LROC NAC data to better individuate the site within the crater at a resolution of roughly 37 m/pixel; the main point of reference for the site is a crater of 1 km in diameter centered at 88.76°S–232.00°W (Figure 2B). We have also shown in Figure 2B the shortest path (yellow line) toward the PSR from the illuminated terrain available to deploy the base. A topographic profile shows a slope of roughly 1.15° along the path between the base and the PSRs (Figure 2C).

De Gerlache is, more or less, the same size as Sverdrup (∼33 km, rim-to-floor ∼1.1–3.0 km) but has a more rugged floor. This is largely due to the presence of a younger impact crater (∼15.5 km, rim-to-floor ∼1.0–1.2 km, flat ∼47 km^2^ floor) in its north-western parts. The remaining floor has an area of ∼76 km^2^.

The ∼21 km Shackleton has the sharpest and highest rim of the studied craters, making it also the youngest. It is also the smallest but has an impressive rim-to-floor difference of ∼3.5–4.2 km. Its floor is rugged and has an area of ∼37 km^2^.

Malapert Massif is a ridge ∼120 km from the South Pole along the main meridian. The top of the ridge forms a narrow plateau ∼4.5–5.5 km above the surrounding plains. The beeline distances from there to the closest rims of de Gerlache, Shackleton, Henson, and Sverdrup are ∼115 km, 120 km, 135 km, and 155 km, respectively.

### Water ice

Spectral signatures of the possible presence of water ice were detected from the M^3^ data in all the studied craters, with a particularly dense cluster of ice exposure points on the Sverdrup-Henson floor compared to our other studied locations; 100 points are located on the floor while 31 points are distributed along the walls. Considering only the points in the accessible floor area, verified through available topographic data, we see a concentration of ∼0.21 points per km^2^ inside Sverdrup-Henson, inside Shackleton (6 points on the floor and 32 along the walls for ∼0.16 points per km^2^), and inside de Gerlache (3 points on the floor and 11 along the walls for ∼0.024 per km^2^), all of which have PSRs (Li et al.^8^). Thus, according to our count, the Sverdrup-Henson site contains a total amount of 131 points where the presence of water has been identified (M^3^, [Li et al.^8^]) while de Gerlache and Shackleton craters contain 14 and 38 points, respectively (Figures 1 and 2A). Furthermore, Sverdrup-Henson shows possible access to another unnamed crater located on the west side of its rim (center 88.55°S, 183.0°W), which we have provisionally designated as “Unnamed Crater”. This crater has at least 40 points on its floor. There are also 7 additional points on the external rim of Sverdrup on the way to the Unnamed Crater. With a total amount of 47 points for this area, it is more than the Shackleton crater (38 points) (Figures 1 and 2A).

We have shown the first settlement’s proposed location, either robotic or human, identified as a red square in Figure 2A, where a partially illuminated area exists (Figure 2B). We have also indicated in Figure 2A the location of a possible initial settlement. The path from the base to the PSRs, where the water ice is detected, is indicated in Figure 2B.

### Mineral and elemental resources

Using the combined Kaguya SP-LRO LOLA dataset, we have identified that Sverdrup-Henson also shows a slightly higher abundance of iron (FeO) than the craters de Gerlache and Shackleton (Figure 3). However, the difference is so small (within 1–2 wt % at the resolution of 100 m/pixel) that it can be essentially considered similar and sufficient for the purposes of a human or robotic settlement. Thorium maps obtained from the gamma-ray spectrometer on-board Lunar Prospector showed concentrations between ∼9.5 and 4.1 ppm at the lunar South Pole.^53^^,^^79^ There is the potential to transmute thorium to U-233 *in situ* and to build 2 MW fission piles for a first-generation baseload power plant.^80^ The derived concentration of rare earths (Sm in particular) was estimated to be around 9 ppm as well, mostly found inside impact craters.^79^ However, the scarce olivine detected in the lunar South Pole suggests that the area was originally olivine poor.^81^^,^^82^

### Slope

We analyzed the LOLA slope map over the craters Sverdrup-Henson, de Gerlache, Shackleton, and Unnamed Crater at a resolution of 100 m/pixel.^29^ For additional details, we used the SLDEM2015.^70^ In the following, if the slope angle along a particular route is clearly below 15°, we define it as *navigable* or *accessible*. If the route has short sections with ∼15° slopes, it is deemed *potentially navigable*, pending better resolution data from eventual ground truth observations from rovers and astronauts. Accessibility and navigable distance are listed in Table 1.

**Table 1:** Scheme of accessibility and illumination availability

Solar panel array location	Illumination %	Coordinates	Accessible from the base (with navigable distance)
Henson N rim	50%–55%	From 87.86°S, 130.56°W to 87.75°S, 130.08°W	Sverdrup-Henson (∼45 km)
De Gerlache W-NW rim	50%–64%	88.21°S, 105.92°W (up to 87.99°S, 92.87°W)	Sverdrup-Henson (∼35 km) de Gerlache∗∗; from other end
De Gerlache S rim	50%–79%	88.78°S, 107.59°W (up to 89.02°S, 92.01°W)	Sverdrup-Henson (∼50 km) Shackleton de Gerlache∗
De Gerlache N-NE-E rim	50%–77%	88.04°S, 77.15°W (up to 88.71°S, 68.52°W)	de Gerlache∗∗
Henson SSE rim	50%–79%	89.18°S, 109.27°W (up to 89.32°S, 114.13°W)	Sverdrup-Henson (∼40∗ km) Shackleton
Henson SSW rim	50%–88%	89.53°S, 141.41°W (up to 89.11°S, 151.94°W)	Sverdrup-Henson (∼20∗ km) Shackleton; from the other end
Shackleton rim	50%–87%	89.92°S, 143.97°W (up to 89.66°S, 165.76°W)	Shackleton
Malapert Massif	50%–82%	e.g., 86.00°S, 2.06°E	Malapert Massif

Accessible locations of solar panel arrays around the studied sites. The percentage indicates the portion of time the Sun is above the local horizon; kilometers are navigable driving distances from the base to the array; distances with stars (∗) depict potentially navigable routes, i.e., with some ∼15° slopes. Two stars (∗∗) indicate that access requires traversing through a PSR. Illumination percentages were measured using LROC Quick Map, see Mazarico et al.^7^

The floor of Sverdrup is very flat, generally with <5° local slopes (Figure 4). An easily navigable ∼0.31° slope leads from the base toward the PSR of Henson crater in the southwest (Figure 2C). Sverdrup is additionally connected with another easily navigable <5° slope to the floor of Henson (Figure 4) and with a ∼2.45° slope toward the PSR of the Unnamed Crater in the east (Figure 2D). A wide, easily navigable “main access path” (see the red path in Figure 2A to Henson) and a “secondary access path” (see the blue path in Figure 2A to Sverdrup) both lead from/to the base from/to the intercrater plains in which we have identified a flat area located at a safe distance of ∼30 km (from launch blasts) where it is possible to place a launch pad (Figure 2A). The traversability of this route needs to be verified with more accurate measurements of the local slopes and cratered terrain by prior visitation by rovers and astronauts.

Most of de Gerlache’s floor slopes are <5°, but 7–10° sections also exist. No navigable paths connect it with the floor of the ∼15.5 km-diameter younger crater. De Gerlache is accessible from the north, on the side opposing the highest peaks, but this path requires driving through or just at the margin of a PSR-specific area. Narrow alternative potential paths just at the 15° limit may connect the floor to the antenna/solar panel installations at the southern rim (Figure 2A), as well as to the western rim toward Henson.

Generally, the circular route at Shackleton crater is < 1 km wide, though some ∼100 m wide parts may be deemed too hazardous to pass. Several accessible routes extend from the rim crest toward craters Henson, Sverdrup, Slater (−88.08°S, 111.3°E), and one potential path toward de Gerlache. The crater walls are too steep (≥20°) for navigable paths between the rim crest and the PSR within the crater.

Although it is uncertain how steep slopes would be suitable for lunar base construction, it is safe to say that the potential for base growth on top of the Malapert Massif is much smaller than at the other sites (see Table 3). The potential operative area on top of Malapert Massif is from 12 km^2^ (with slopes <7°) to ∼65 km^2^ (slopes <15°). Additionally, a single ∼45 km long, and only possibly traversable route with short sections just above the 15° limit, leads from the top along the ridge’s western extension to the surrounding intercrater plains. From there, the closest accessible PSR and some water detection points are at least 10 km away (Figure 1).

Even though the current altimetry data do not have high enough resolution to determine the local terrain slopes at the lander footprint scale, we find that the average regional slope of the floor of the three crater sites is < 15° (Figure 4), i.e., within the upper limit above which a certain surface is considered hazardous for a lander.^83^^,^^84^ The floor of the Sverdrup-Henson site is characterized by the most gentle slopes, mostly <5°. Although the resolution of the LOLA dataset does not allow the recognition and selection of specific landing sites in this crater, this result is encouraging and provides a starting point for more detailed ground investigations.

### Illumination conditions

We reviewed the conditions of illumination within Sverdrup-Henson and surrounding craters using Kaguya data.^71^ Henson and de Gerlache craters share a rim, which, similarly to the massifs between de Gerlache and Shackleton, is in places illuminated for more than 80% of the year, where 0% means total darkness or no visibility and 100% is fully visible/illuminated (Figure 5). The best-illuminated locations are on the eastern rim of de Gerlache, the massifs located between the craters de Gerlache and Shackleton, and the eastern rim of Shackleton.^7^^,^^38^^,^^71^ The Henson crater has some illumination, around 15%, during the lunar day.

### Communications with Earth

Similarly to the illumination, we made second-order approximations on how communications with Earth might be organized at the various base sites, again taking slopes into consideration for maintenance and installation traverses. Results are detailed in Tables 1 and 2.

**Table 2:** Scheme of Earth visibility

Communication antenna location	Earth visibility %	Coordinates	Accessible from the base site (with navigable distance)
Henson N rim	43%	87.85°S, 130.74°W	Sverdrup-Henson (∼45 km)
De Gerlache rim	57%	88.00°S, 97.03°W	Sverdrup-Henson (∼50 km)
De Gerlache S rim	59%	89.02 S, 92.08 W	Sverdrup-Henson (∼50∗ km) Shackleton de Gerlache∗
De Gerlache N rim	60%	87.99°S, 91.17°W	Sverdrup-Henson (∼55 km) de Gerlache∗∗
N of de Gerlache	58%	87.94°S, 82.40°W	de Gerlache∗∗
Henson SSW rim	58%	89.44°S, 136.60°W	Sverdrup-Henson (∼35∗ km) Shackleton
Shackleton rim	57%	89.79°S, 155.60°W	Shackleton
Malapert Massif	100%	Several	Malapert Massif

Locations where the Earth is visible for extended periods and thus accessible from the studied sites. The percentage indicates the portion of time Earth is above the local horizon; kilometers are navigable driving distances from the base to the array; distances with stars (∗) depict potentially navigable routes, i.e., with slopes around 15°. Two stars (∗∗) indicate that access requires traversing through a PSR. Earth visibility percentages taken from LROC Quick Map, see Mazarico et al.^7^

The maximum values of Earth visibility obtainable from each of the three crater sites are only 40%–60%^7^ (Figures 4 and 5), which is probably too small for at least robotic missions. In such cases, alternative communication methods must be used. The problem of the lunar curvature can easily be overcome by (1) installing a series of wireless signal repeaters along the line of sight shown in Figure 2A in order to maintain the continuity of the signal, or (2) deploying a Moon-orbiting relay satellite network.^85^ However, further studies are required to determine what kind of relay satellite network would be needed and how their orbits will change over time. In any case, the wireless signal repeaters should solve the problem.

## Discussion

Given the presented criteria, we discuss the pros and cons between our crater candidates at the lunar South Pole in order to constrain the best possible compromise in the selection process for a first settlement on the Moon.

Each location has a certain degree of potential (see Table 3); however, we consider the site at Sverdrup-Henson much better than the other locations in terms of resources, ease of access, crater slope, the flexibility of Earth communication, and the possibility of solar power from nearby illuminated areas. Its main strength is its vicinity to the water resources in the PSR and to the illuminated areas close to the rims of the nearby craters de Gerlache and Shackleton.

**Table 3:** Scheme of water availability

Characteristics/Sites	de Gerlache	Sverdrup-Henson	Shackleton	Malapert Massif
The main site (area)	Crater floor (∼76 km^2^)	Crater floor (∼479 km^2^)	Crater rim crest (?)	Ridge top (<65 km^2^)
Inaccessible nearby sites of interest	Small crater floor	–	Crater floor	All lower areas except in the west
Accessible paths outside the main site (and potentially accessible paths)	1 north (1 south; 1 west)	1 north (1 southwest; 1 south) 1 west 1 east	Numerous; to various directions	0 (1 west)
Presence of PSRs (area)	Yes (235 km^2^)	Yes (545 km^2^)	Yes (19 km^2^)	No (nearest 50 km away; 13 km^2^)
Spatial density of water ice occurrence M^3^ data	Scarce (14 points)	Excellent (131 points)	Adequate (38 points; not accessible)	–
Iron oxide occurrence	Sufficient (No discernible difference between the sites)			
Illumination	Good (<80%)	Very good (<65%–90% 20–50 km away)	Very good (<90%	Good (<80%
Earth visibility	Fair (<60%)	Fair (43%–59% 45–70 km away)	Fair (<60%)	Excellent (100%)

Comparison of criteria between Sverdrup, de Gerlache, and Shackleton craters and Malapert Massif. See sections Methods for the assessment of landing site suitability criteria and Results for further details.

Crater de Gerlache has fewer ice exposure points than the other craters, whereas Shackleton shows a sufficient number of points, but only on its eastern side. Furthermore, both craters have high and steep rims with no passes that allow easy access to their resources. Although the slope characteristics of the floor of the three craters are acceptable and within limits imposed by the safety criteria for landing and rover operations, access to Sverdrup-Henson is by far the easiest and most beneficial. Sverdrup-Henson also shows a larger flat-floored surface than the other craters, allowing a future expansion of the base if needed. Analysis of iron oxide abundance data did not reveal any dramatic differences between the candidate sites (Figure 3).

Although Sverdrup-Henson is the less efficient candidate in terms of obtaining solar power and keeping near-constant communication with Earth, the rims of Shackleton and de Gerlache are higher than those of Sverdrup or Henson and they are connected to a massif with similar properties.^7^^,^^38^^,^^71^ This massif area is best suited for solar panel installations, and placing antennas, possibly through a relay antenna chain placed on top of the Malapert Massif (see Sharpe & Schrunk^1^). Placing the settlement on the de Gerlache-Shackleton rims or the connecting massif would thus improve the energy and communication conditions, while at the same time reducing the accessibility to low-lying resources. We propose that a settlement within Sverdrup-Henson could exploit the high elevations of this connecting massif by using wireless electric power through the available lines of sight (white lines in Figure 2). In our view, the proposed Sverdrup-Henson site in Figures 2A–2C is a suitable trade-off between access to resources, and energy and communications requirements (Table 3).

A future base will nevertheless need both types of locations, including the various other criteria described so far.

### Future lunar exploration and objectives

The Apollo missions have generally landed on the safe surfaces of the lunar maria, which are characterized by mafic composition, thus underrepresenting the anorthositic composition of the lunar highlands in the samples collection. Now, with a possible series of missions to the lunar South Pole, there is the opportunity to cover this gap and thus study the formation and evolution of lunar regolith at high latitudes along with the trapping mechanisms of volatile compounds in the PSRs. The prospect of an eventual human settlement on the Moon is relevant to the broader lunar community, as stated in the Advancing Science of the Moon: Report of the Lunar Exploration Analysis Group (LEAG) Special Action Team document.^86^ Concept #4 of this report has prioritized the lunar poles for being a special environment for understanding the volatile constituents observed at the lunar poles. Also, Concept #7 states that the Moon is “a natural laboratory for regolith processes,” emphasizing our need to create a working environment on the lunar surface to study the regolith and utilize resources.

The Sverdrup-de Gerlache region has been designated as a target of interest for the Artemis III mission.^87^^,^^88^ Such a landing area (designated as “Connecting Ridge”, “Connecting Ridge Extension”, “de Gerlache Rim”, and “de Gerlache Rim 2”) would have numerous resources and opportunities to investigate the frozen volatiles within the area for not only science priorities (i.e., the origins and chemical makeup of the volatiles; see Stopar and Shearer^23^) but also data for future explorations to use such chemicals for fuel and base-camp construction. The Artemis III Science Definition Team Report has also stated several priorities of using the lunar surface for eventual settlement and utilizations thereof, such as (i) Goal 1F and 1F-1: regolith processes and weathering, and to determine physical properties of regolith at diverse locations of expected human activity; (ii) Goal 2b and 2c: determine the source(s) for lunar polar volatile deposits and understand the processes of volatile materials; (iii) Goal 2d: understand regolith modification processes; (iv) Majority of Goal 6 is to use the lunar environment to test regolith material for eventual habitats and the management thereof.

Other lunar missions will also be advantageous for eventual settlement objectives and missions. While future National Aeronautics and Space Administration (NASA) Commercial Lunar Payload Services (CLPS), like Peregrine and Blue Ghost, and other international missions, such as the Korea Pathfinder Lunar Orbiter, are slated to venture to the Moon in the next decade across various locations on the lunar surface, these will also be useful to understanding the regolith properties to use for human settlement and habitat-building, and especially establishing a communications network to eventually be used by multiple astronautic facilities.

### Possible instruments for ISRU at Sverdrup-Henson

Confirming orbital observations with ground truth should be a priority task for future landers near the lunar South Pole. The priority task includes *in situ* detection and quantity estimation of water ice (including within PSRs and within the lunar regolith) and mineral resources, which is particularly important for the NASA Artemis program and its planned landing of astronauts at the South Pole in 2024.^89^ Therefore, it is necessary to take a closer look at sunlit circumpolar regolith properties that may ultimately affect landers, infrastructure, and robotic (and eventually crew) traverses.

The first mission to investigate and sample *in situ* lunar polar materials is the Volatiles Investigating Polar Exploration Rover (VIPER) mission,^90^ developed through NASA’s Lunar Discovery and Exploration Program (LDEP) proposed to fly in late 2023. The main purpose of the VIPER rover would be to (i) locate surface and subsurface volatiles; (ii) excavate and analyze samples of volatile-bearing regolith; and (iii) demonstrate the extractability of the materials. Its instruments include a neutron spectrometer for remote subsurface hydrogen (and water ice) detection, a drill for sampling the topmost 1 m of regolith, and a near-infrared spectrometer and a mass spectrometer for analyzing the mineral and volatile composition of the obtained samples. Such objectives and instrumentation lead to (i) ground truth for orbital datasets and models; (ii) correlation of surface environments and volatiles with orbital datasets for future resource mapping; and (iii) identify polar volatile retention and distribution for future excavation, which would be useful for the NASA Artemis program.

A rover similar to VIPER at Sverdrup would provide the means to study this PSR crater and close proximity to other PSR sites, which could lead to further hypotheses of how volatile distribution and chemical processes may differ across various PSRs. Rover operations at Sverdrup also bring some challenges, such as low temperatures, localized steep slopes at the crater walls, and potential boulder obstacles.^91^ Mapping of challenging terrain (e.g., sinkage of the VIPER rover up to ∼4 cm with slopes >25°)^89^ has been made to compare potential south polar PSR craters. Of note is that Sverdrup shows a “potentially challenging” region to the northeast of its rim (slopes >25°), but otherwise less challenging (slopes <15°) between the Henson and Sverdrup. In comparison, Unnamed Crater has a steep terrain in its rim vicinity, and de Gerlache also has a challenging terrain at its rim that extends to the southeast. From this assessment, the region between Henson and Sverdrup offers the least slope challenges regarding traverse planning.

The concept of a Lunar Volatiles Scout (LVS) drill mounted on a Polar Ice Explorer (PIE) rover, with a total mass <16 kg, able to traverse slopes of 15° (but tested for 20°), and equipped with solar cells providing a maximum of 35 W for charging the Li-batteries and operation supply, including thermal passivization for the survival of the equipment at very low temperature, has been designed to overcome the challenges related to the harsh environment of the PSRs.^92^ The technical feasibility of the ground operations within 2 km radio-signal range in the PSR of the Amundsen crater has been simulated for a 10 days illumination period for battery recharge with a planned mission duration of 161 h; 15 W of heater power is optimal to extract >80% of regolith water after 90 min, and consumption reaches a maximum of 50 W during drilling operations and a minimum of 13 W when the rover is in safe mode.^92^ This performance can be improved with available solid-state batteries with reduced net weight, greater and more uniform energy output, reduced volume,^93^^,^^94^ and increased low-temperature tolerance.^95^ These advanced batteries can further benefit from a coupled power extension through available radioisotope thermal generators (RTGs); current Multi Mission RTG (MMRTG) can generate about 110 W.^96^ Advanced Li-batteries can reach operating temperatures as low as 203 K (−70°C),^95^ but solid-state batteries using silver as electrolyte can reach 233 K.^97^ The performance of the Li-battery can be improved to 173 K (−100°C) using sulfolane-based (SL) with medium LiFSI concentration (1–1.5 mol dm^−3^) electrolytes; such a progress is ascribed to the noncrystallinity of the electrolyte although further measures are needed to improve electrochemical performance at lower temperatures.^95^ Other “novel concepts for energy storage include flywheel devices that store energy in a mechanical form or superconducting inductive loops that could take advantage of the natural vacuum and cryogenic temperature conditions in shadowed areas.”^96^ Of course, these data come from simulations made on Earth, but further tests should be conducted directly on the PSRs of the Moon for confirmation.

### Conclusions

Thanks to the combined datasets of various missions, a preliminary analysis of a possible settlement site near the South Pole of the Moon is now available. Such a settlement could be built inside Sverdrup-Henson (center 88.5°S, 129.6°W) just downhill from the rim of de Gerlache. Its strategic position, easy access, availability of resources (water ice and minerals), energy, and the possibility of communications with Earth make Sverdrup-Henson a potentially suitable location for establishing the first settlement at the lunar South Pole. A potential next step would be to investigate the chemical content of the Sverdrup-Henson Unnamed Crater site on the surface and at shallow depths with a lander to verify orbital observations and better assess the actual quantity of water ice present. At last, although the Henson-Sverdrup area is potentially the best place for the first settlement, we will need all the water that we can get from all the sites in case of an extended campaign of colonization of the Moon.

### Limitations of the study

It is imperative to understand whether the spectroscopic detections correspond to some amount of a water reservoir realistic for long-term surface operations. Furthermore, we need to test technologies and long-lasting batteries that would allow rovers and other potential mobile units to operate inside the PSRs under extreme cold and reduced illumination.

## STAR★Methods

### Key resources table

**Table:** 

REAGENT or RESOURCE	SOURCE	IDENTIFIER
Moon Trek	NASA	https://trek.nasa.gov/moon/
JMARS	Arizona State University	https://jmars.asu.edu
PSRA	Arizona State University	https://www.lroc.asu.edu/psr
LROC Quickmap	Arizona State University	https://quickmap.lroc.asu.edu/

### Resource availability

#### Lead contact

Further information and requests for resources and reagents should be directed to and will be fulfilled by the lead contact, Giovanni Leone (giovanni.leone@uda.cl).

#### Materials availability

This study did not generate new unique reagents.

### Experimental model and subject details

Our study does not use experimental models typical of the life sciences.

### Method details

We conducted a preliminary survey using the datasets listed in the key resources table. We imported the datasets listed below, and analysed them using ArcGIS. Measurements of areas, distances, elevations, and slopes (results for which are shown in sections Craters in the area of study and site location and Slope), as well as analysis of accessible sites of maximum illumination and Earth visibility (sections Illumination conditions and Communications with Earth) were made using the LROC Quickmap online tool. Ready-to-use map of M^3^ water detections is taken from the MoonTrek website.

#### Material preparation

Our study does not materials typical in the life sciences.

### Quantification and statistical analysis

There is no statistical analysis in this paper.

### Additional resources

We have no relevant resources.

## Data Availability

The datasets were derived from sources in the public domain: [NASA Moon Trek (https://trek.nasa.gov/moon/); JMARS (https://jmars.asu.edu); Permanently Shadowed Regions Atlas (PSRA) (https://www.lroc.asu.edu/psr); LROC Quickmap (https://quickmap.lroc.asu.edu/); the illumination map of the Moon’s South Pole at 100 meters/pixel shown in Figure 5 was taken from: https://solarsystem.nasa.gov/resources/2329/illumination-map-of-the-moons-south-pole/].
